# Coronary Artery Disease and Severe Aortic Stenosis: Contemporary Treatment Options for Patients Undergoing Transcatheter Aortic Valve Implantation

**DOI:** 10.3390/jcm13247625

**Published:** 2024-12-14

**Authors:** Nikolaos Ktenopoulos, Antonios Karanasos, Odysseas Katsaros, Anastasios Apostolos, George Latsios, Athanasios Moulias, Michael I. Papafaklis, Grigorios Tsigkas, Constantinos Tsioufis, Konstantinos Toutouzas, Periklis Davlouros

**Affiliations:** 1 First Department of Cardiology, National and Kapodistrian University of Athens, Hippokration General Hospital of Athens, 11527 Athens, Greece; nikosktenop@gmail.com (N.K.); odykatsaros@gmail.com (O.K.); anastasisapostolos@gmail.com (A.A.); glatsios@gmail.com (G.L.); ktsioufis@gmail.com (C.T.); ktoutouz@gmail.com (K.T.); 2 Department of Cardiology, Patras University Hospital, 26504 Patras, Greece; dramoulias@live.com (A.M.); m.papafaklis@yahoo.com (M.I.P.); gregtsig@upatras.gr (G.T.); pdav@upatras.gr (P.D.)

**Keywords:** TAVI, TAVR, coronary artery disease, aortic stenosis, cardiac catheterization, transcatheter aortic valve intervention

## Abstract

Approximately 50% of individuals eligible for transcatheter aortic valve implantation (TAVI) have coronary artery disease (CAD). The influence of CAD, both its prevalence and severity, on post-TAVI clinical results has yielded conflicting findings. Recent research has shown positive results for the use of computed tomography angiography and functional percutaneous evaluation of coronary lesions in the pre-TAVI assessment, besides the classic coronary angiography. As we anticipate the outcomes of current randomized studies, it has become common practice to perform invasive revascularization on TAVI patients with obstructive CAD. Furthermore, there is a lack of comprehensive data about the occurrence, features, and treatment of coronary incidents after TAVI. There is also growing concern about the possible difficulties in accessing the coronary arteries in patients who need coronary angiography with or without intervention after TAVI. This review presents a comprehensive analysis of the contemporary treatment options of CAD in patients undergoing TAVI. In this context, it examines the incidence of CAD in TAVI candidates; its clinical significance; the assessment and management of CAD before, concomitant, and after the procedure, including patients’ unresolved concerns; and possible future aspects.

## 1. Introduction

Aortic stenosis (AS) is a prevalent valvular heart condition that becomes more common as the population ages [1,2]. The occurrence of severe AS among the elderly ranges from 3 to 5% [3]. Coronary artery disease (CAD) is prevalent within this group, and the conventional approach to treating severe AS with CAD has been a combination of surgical aortic valve replacement (SAVR) and coronary artery bypass grafting (CABG) [4]. The advent of transcatheter aortic valve implantation (TAVI) has provided another feasible option for patients, who are presented now with the less invasive alternative of combining TAVI with percutaneous coronary intervention (PCI). The evaluation and treatment of CAD in this particular context provide distinct difficulties and factors to take into account due to the intricate physiological and anatomical interactions associated with all the types of AS [5], the transcatheter heart valves (THVs) [6], and the TAVI process [7].

According to the latest international guidelines, it is recommended to perform PCI on coronary artery fragments with more than 70% narrowing in the proximal segments [or more than 50% in the case of left main (LM) disease] in patients who are scheduled for TAVI, based on angiographic evaluation. This recommendation is classified as class IIa, with a level of evidence C [8]. Considering that this recommendation is based on non-randomized, observational studies with inherent limitations, there is still a need to determine the best diagnosis and therapy for CAD in patients who are scheduled for TAVI. The evaluation of the severity of CAD in the presence of AS, in a tricuspid or bicuspid valve [9], the degree of revascularization, and the most appropriate time for both treatment options are still subjects of discussion. Traditionally, before TAVI, it was common practice to perform percutaneous revascularization because of concerns about ischemic and hemodynamic adverse events that may occur during the rapid ventricular pacing. At present, the order in which specific therapies are performed is determined by individualized decision-making, taking into account clinical and anatomical factors. Each timing method and access site has its own potential benefits and drawbacks [10,11,12].

Although the clinical significance is substantial, there is a paucity of sufficient data. Moreover, the applicability of findings from these studies is limited considering that they have small sample sizes, are mainly focused on balloon-expandable valves (BEV) [13], and are focused on specific subgroups. Ongoing randomized studies are now examining the relevance of physiological evaluation of CAD and proper timing methods for revascularization in individuals with severe AS who require TAVI [14,15,16]. Therefore, the objective of this review is to present a contemporary and comprehensive analysis of CAD in individuals undergoing TAVI. This study will specifically address the prevalence of CAD in TAVI patients; the clinical relevance; the evaluation and management of CAD before, concomitant, and after the TAVI procedure, including patients’ unresolved concerns; and possible future aspects.

## 2. Epidemiology and Prognostic Impact

The incidence of CAD has been approximately 50% [17]. In randomized controlled studies, the incidence of CAD has decreased from 81% to 15% due to the inclusion of younger patients with lower surgical risk. Low-risk patients have a significantly lower prevalence of CAD compared to intermediate- and high-risk patients [18,19]. Approximately 50% of TAVI candidates with CAD have multivessel disease, while a recent study of 4000 TAVI recipients with CAD found that the average SYNTAX score (SS) was around 14. In this series, 11% of patients had LM disease and one-half had left anterior descending (LAD) disease [20].

In recent times, large registries have provided contemporary empirical evidence. The UK and German TAVI registry studies [21,22], along with a trial conducted at the University Hospital of Toulouse, found no evidence to imply that preceding CAD affected mortality; on the other hand, a TAVI registry conducted in Bern, Switzerland, reported a higher occurrence of ischemic incidents and cardiovascular-related deaths, after 1 year, in patients with CAD compared to those without CAD (HR 1.75; 95% CI [1.06–2.89]; *p* = 0.030) [23]. Furthermore, a meta-analysis conducted in 2017 included 8013 patients who underwent TAVI using either a self-expanding valve (SEV) or BEV [24]. The study found that there was no increase in death within 30 days but there was an increase in overall mortality after 12 months (OR: 1.21; 95% CI [1.07–1.36]; *p* = 0.002). The conflicting results are in line with the diverse characteristics of CAD and can be attributed to the varying criteria for inclusion in each study and the varying levels of ischemia seen in them. This is not surprising, given the previous data in CAD without AS, which have demonstrated that managing ischemia is beneficial.

## 3. Pre-Procedural Assessment of CAD

Major recommendations strongly advise evaluating for CAD prior to performing aortic valve intervention. This is because of the high frequency of CAD, its significant influence on prognosis, and the possible challenges that may arise in accessing the coronary arteries in the future. Prior to making a choice on a combined SAVR and CABG, the heart team should take into account the intricacy of CAD, the surgical risk, and the aortic root along with the vascular architecture [11,12]. A higher level of CAD complexity—such as LM or multivessel CAD with a high SS or a high-risk aortic root architecture—may indicate that a combined strategy of SAVR and CABG is more advantageous compared to TAVI and PCI [25].

In high-risk TAVI patients, invasive coronary angiography is routinely performed first to assess for CAD because of its high prevalence. As the use of TAVI expands to cover low-risk patient groups, the probability of CAD in these individuals may decrease. Consequently, the ratio of risk to benefit for an invasive operation may be altered. Computed tomography coronary angiography (CTCA) has demonstrated sufficient accuracy in excluding severe CAD in younger individuals with little coronary calcification [26]. CTCA has the added benefit of being able to be combined with the standard CT examination performed before TAVI. This may be performed without the need for extra contrast, which assists in avoiding intrusive assessments and any hazards associated with the procedure. The potential influence of CAD and the role of revascularization in individuals undergoing TAVI have remained uncertain following the diagnosis. Preliminary trials have yielded inconsistent findings, possibly due to the diversity of the population studied and the small sample sizes used. The results have ranged from an increased risk of death following TAVI to no significant effect [27].

Lately, recent evidence has challenged the contemporary practice in many centers of performing an invasive coronary angiography as part of the assessment for TAVI. This is due to the high diagnostic accuracy of the CTCA in excluding significant CAD. Current CAD recommendations endorse both the CTCA as a screening examination and the systematic utilization of hemodynamic functional evaluation methods for assessing the need for revascularization [8]. Ongoing trials aim to enhance our understanding of the value of these technologies within the context of TAVI. With the growing acceptance of TAVI as a viable therapeutic alternative for younger patients with lower risk and longer life expectancy, the management of concurrent CAD is becoming increasingly prominent [10].

The current recommendations for chronic coronary syndromes (CCS) suggest that invasive functional assessment should be performed using either fractional flow reserve (FFR) or instantaneous wave-free ratio (iFR) to decide for revascularization when there are coronary lesions present in the myocardial region without any signs of ischemia [28]. In addition, an observational trial conducted by Lunardi et al. showed that using FFR guidance for PCI in individuals undergoing TAVI with concurrent CAD resulted in better cardiovascular and cardiac-event-free survival rates at 24 months compared to a strategy of revascularization guided by angiography [29]. Moreover, several studies have demonstrated the safety of FFR evaluation in individuals with severe AS, despite the use of intravenous or intra-coronary adenosine. The current FAITAVI study aims to compare the efficacy of angiography-guided versus physiology-guided strategies in individuals with severe AS and substantial CAD [16]. This study may provide more evidence for the need for hemodynamic functional evaluation in candidates for TAVI. An important drawback of FFR in this situation is the possibility of coronary flow reserve being affected by the LV hypertrophy, a condition often observed in severe AS. This might lead to an underestimation of the extent of coronary stenosis. Resting pressure indexes, like iFR, evaluate the pressure ratio during the wave-free period of diastole. These indexes may address the issue of compromised microvascular function seen in AS. They seem to be less affected by the coronary flow reserve and do not require the use of a vasodilator.

The recent NOTION-3 trial [14] specifically evaluated the outcomes of combining FFR-guided PCI with TAVI in patients with severe AS and stable CAD. The study randomized 455 patients scheduled for TAVI with at least one coronary stenosis with FFR < 0.80 or diameter stenosis > 90% to PCI and TAVI or isolated TAVI. The combination of PCI and TAVI significantly reduced the composite outcome of all-cause mortality, myocardial infarction, and urgent revascularization versus isolated TAVI (26% versus 36%; *p* = 0.04); however, the PCI group experienced significantly higher rates of minor bleeding (28% versus 20%). Despite the bleeding risk, the researchers suggested that PCI should be considered in patients with significant coronary lesions undergoing TAVI, with decisions individualized based on the patient risk factors and the lesion severity. As a result, these findings offer valuable insights for improving the management of CAD in TAVI patients.

Furthermore, assessing myocardial viability has emerged as a potential tool in the decision-making process for PCI in patients undergoing TAVI. Myocardial viability testing, utilizing modalities such as cardiac magnetic resonance (CMR), positron emission tomography (PET), or single-photon emission computed tomography (SPECT), provides insights into the presence of hibernating or ischemic myocardium that might benefit from revascularization [30]. This assessment can guide clinicians in determining the necessity and timing of PCI, particularly in patients with concomitant CAD and severe LV dysfunction. In the context of TAVI, where patient selection and procedural outcomes hinge on a careful balance of risks and benefits, myocardial viability testing can refine the identification of candidates who are most likely to derive clinical benefits from PCI. By differentiating viable myocardium from irreversibly scarred tissue, the viability assessment aids in avoiding unnecessary interventions that might expose patients to additional risks without improving outcomes [30].

CMR imaging is a powerful tool in this context, capable of assessing myocardial perfusion, viability, and proximal coronary anatomy, as well as providing prognostic insights into the extent of myocardial fibrosis and LV remodeling. Evidence from a small study demonstrated high sensitivity and specificity for stress-perfusion CMR compared to invasive coronary angiography in patients with severe AS, with adenosine stress perfusion performed safely even in this high-risk population [31]. Despite its advantages, the use of CMR in the pre-TAVI pathway is technically demanding and should not replace more streamlined modalities like MSCT. Instead, CMR should be reserved for specific indications, such as evaluating complex CAD or unclear ischemic burden. Integrating myocardial viability data into clinical decision-making ensures that PCI is offered only to patients likely to benefit from revascularization, avoiding unnecessary risks while optimizing outcomes. Further studies are warranted to standardize its application in this unique cohort.

## 4. Role of Coronary Revascularization in TAVI Candidates

The clinical significance of performing PCI during the work-up for TAVI, as an elective or an urgent procedure [32], has yet to be established. Randomized studies have shown that PCI does not have a clear positive impact in patients with stable CAD. Current recommendations emphasize that there are insufficient scientific data supporting the use of PCI when there is a stenosis of more than 70% in the proximal segments of a coronary artery [8].

Multiple observational studies have demonstrated no significant differences in clinical outcomes between patients who receive TAVI + PCI and those who just undergo TAVI without any other intervention [17,33]. Furthermore, a recent meta-analysis has indicated that performing PCI prior to the THV procedure does not result in an elevated risk of mortality within one year [34]. These findings have served as evidence supporting the feasibility and safety of revascularization in this scenario. Furthermore, several trials have investigated the completeness of revascularization in individuals eligible for TAVI who also have CAD, predominantly by assessing the residual SS (rSS). Multiple studies have demonstrated the adverse effects of incomplete coronary revascularization and/or high rSS, even after accounting for the potential confounding factors [35,36]. These findings are also bolstered by two recent meta-analyses that validate the fact that patients with elevated rSS experience a greater death rate following TAVI [37,38]. Nevertheless, the decision regarding which coronary lesions to target and the extent of PCI was typically determined by the interventional cardiologist or heart team at each hospital and could have been regulated by additional factors such as angina or reduced LV ejection fraction (EF). This resulted in significant variation in the PCI strategies across the studies, which could have potentially affected the clinical outcomes.

Despite the lack of consistent evidence supporting a positive impact of routine coronary revascularization in patients with CAD scheduled for TAVI, current guidelines advise performing PCI for TAVI candidates with substantial CAD nevertheless [8]. The ACTIVATION study, the first prospective randomized controlled trial investigating the subject, demonstrated no significant difference between the PCI and no-PCI groups in terms of the primary composite endpoint of death or rehospitalization at one year [39]. Mortality was similar in both groups, and there was no statistically significant difference in major adverse cardiovascular or cerebrovascular events (MACCE). However, the PCI group experienced a higher incidence of bleeding, driven by the increased use of DAPT. However, the ACTIVATION trial had several significant shortcomings that impacted its findings [39]. The trial was prematurely terminated, enrolling 235 patients instead of the planned 310, reducing its statistical power to detect differences between the PCI and no-PCI groups. The unblinded design could have introduced bias, and the higher-than-expected rate of bleeding events in the PCI arm was likely influenced by increased dual anti-platelet therapy (DAPT), further complicating interpretation. Furthermore, the short follow-up of one year limits the ability to assess long-term outcomes, particularly in elderly patients with complex coronary disease. The negative results of the trial give rise to concerns about the associated risks, particularly bleeding, highlighting the need for individualized treatment strategies based on patient risk profiles.

Additional insights regarding the role of revascularization in patients scheduled for TAVI are provided by the multicenter REVASC-TAVI registry [22]. Propensity score matching was used for the comparison of outcomes between patients undergoing incomplete and complete revascularization. The comparison, based on 657 matched pairs of patients, demonstrated no significant differences in all-cause mortality and the composite of death, stroke, myocardial infarction (MI), and heart failure rehospitalization between patients who underwent complete versus incomplete revascularization. These outcomes should be interpreted carefully, considering a potentially large selection bias considering that the decisions for revascularization were made based on Heart team consensus, without consistent criteria. Additionally, incomplete data on symptom follow-up and anti-thrombotic therapy make it difficult to assess long-term outcomes comprehensively. Nevertheless, the results of the trial suggest that a more conservative approach regarding revascularization may be considered in elderly patients undergoing TAVI, emphasizing the importance of individualized treatment strategies. Overall, available data regarding the role of revascularization in patients with CAD scheduled for TAVI are rather limited and contradictory, highlighting the need for further randomized studies that can better define the role of revascularization in TAVI patients and identify the patients more likely to benefit.

On top of that, randomized studies have compared TAVI with SAVR for the treatment of individuals with severe symptomatic AS who have low-to-moderate surgical risk (Table 1) [25,40,41]. These trials have included patients who also had CAD. All patients with CAD who participated in these studies were eligible for either PCI or CABG. Patients who were assigned to TAVI underwent PCI in the weeks leading up to the surgery, while those assigned to SAVR underwent CABG at the same time. Approximately 12% of the patients included in the study had revascularization in the coronary arteries, either by PCI or CABG, in addition to TAVI or SAVR. The range of patients who underwent coronary revascularization was between 4% and 22%. The overall findings from all randomized studies demonstrated that TAVI was either not inferior or better than SAVR concerning the combined outcome of death or stroke during the mid-term follow-up interval of 12 to 24 months. While individuals with CAD who needed revascularization were not the majority, these findings support the feasibility of an approach combining TAVI and PCI instead of SAVR + CABG for treating AS and CAD [41,42]. The recent PARTNER 3 study was the only trial reporting specific data on patients who underwent coronary revascularization, either by PCI or CABG. The trial revealed a lack of difference in the outcomes of patients who received TAVI combined with PCI (*n* = 32) and those who underwent SAVR combined with CABG (*n* = 58) [43]. The combined risk of death, stroke, and rehospitalization was 9.4% in the TAVI + PCI group, compared to 12.1% in the SAVR + CABG group. Of note, peri-procedural MI rates were not reported. Furthermore, randomized studies excluded individuals with complex CAD (including involvement of the LM artery or an SS greater than 22 or 32). Chakravarty et al. recently presented data that support the safety of performing LM PCI in TAVI patients [21]. Additionally, some researchers have supported the possibility of utilizing mechanical support devices like Impella (manufactured by Abiomed, Danvers, Massachusetts) or TandemHeart (manufactured by TandemLife, Pittsburgh, Pennsylvania), or conducting balloon aortic valvuloplasty (BAV) before the complex PCI in individuals with severe AS. Further research is required to ascertain the clinical results in cases with complex CAD receiving PCI combined with TAVI.

To sum up, the management of severe CAD in patients undergoing TAVI remains an area of limited evidence and ongoing debate. Despite the growing use of TAVI in patients with severe AS with concomitant CAD, there is a significant gap in understanding the optimal revascularization strategy. Current guidelines [8] offer some direction, recommending PCI for significant proximal CAD in TAVI patients (Class IIa), yet these recommendations are largely based on expert consensus and observational studies rather than robust randomized controlled trials. One of the primary challenges is determining whether complete revascularization improves clinical outcomes or if a more conservative approach is sufficient. Existing data from studies like the ACTIVATION trial [39] and the REVASC-TAVI registry [22] suggest that complete revascularization may not offer significant benefits in terms of mortality, stroke, or myocardial infarction compared to incomplete revascularization, while results from the NOTION-3 study [14] support a favorable impact of functionally complete revascularization. However, these studies have their limitations, including small sample sizes or a non-randomized design. As a result, the lack of strong evidence underscores a critical need for further research, particularly randomized trials, to address key unanswered questions. These include the role of physiological lesion assessment using tools like FFR or iFR, the timing of PCI relative to TAVI, and the long-term impact of revascularization on outcomes such as heart failure and quality of life. Until these gaps are addressed, clinical decision-making will continue to rely on individualized risk assessment, balancing the procedural risks of PCI with the potential for improved coronary flow and reduced ischemic events in high-risk TAVI patients.

## 5. Optimal Timing of Coronary Revascularization

**Figure 1:**
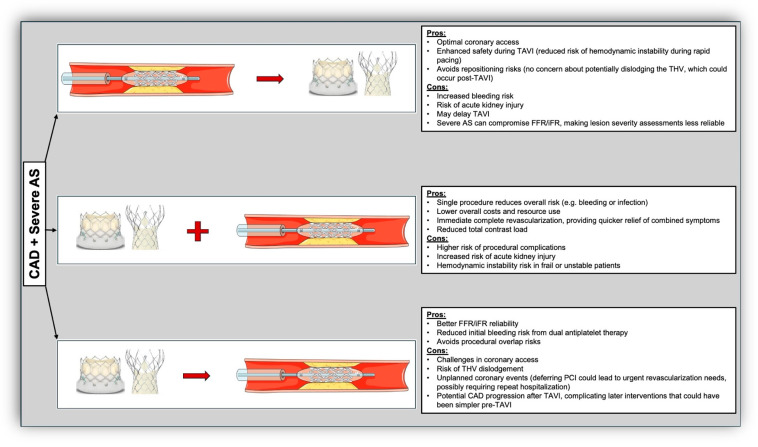
**Central illustration.** This figure delineates the potential benefits and limitations of the three strategic approaches to revascularization in managing CAD among patients undergoing TAVI: pre-TAVI, concomitant with TAVI, and post-TAVI. Pre-TAVI revascularization allows for CAD stabilization prior to valve intervention, potentially mitigating peri-procedural complications, though it may delay TAVI. Concomitant revascularization synchronizes CAD and valve interventions, optimizing procedural efficiency but at the cost of increased complexity and risk of complications. Post-TAVI revascularization simplifies the initial procedure, prioritizing valve implantation but may defer CAD management, posing risks of delayed adverse events and challenges in coronary access. Abbreviations: CAD: coronary artery disease, AS: aortic stenosis, TAVI: transcatheter aortic valve implantation, THV: transcatheter heart valve, FFR: fractional flow reserve, iFR: instantaneous wave-free ratio, PCI: percutaneous coronary intervention.

Table 1, Table 2 and Table 3 summarize all the completed and ongoing trials comparing different treatment strategies for CAD management and revascularization in patients with severe AS undergoing TAVI. Figure 1, the central illustration, summarizes all the advantages and disadvantages of the three revascularization strategies. There are currently no conclusive data about the optimal time of PCI in patients undergoing TAVI who also have severe CAD. When a PCI indication is established, it is often conducted prior to TAVI. However, available options also include the possibilities of simultaneous PCI and TAVI procedures and performing PCI after the TAVI procedure. Performing a staged PCI before TAVI unavoidably involves an extra vascular puncture, several injections of contrast media, and the use of DAPT. These factors might potentially raise the risk of complications after TAVI. Moreover, there is ongoing debate regarding the optimal time interval between the two procedures. Currently, it is not possible to suggest a precise time approach in this context, due to the lack of available data. Nevertheless, it is advisable to consider the coexistence of intricate CAD and risk factors for contrast nephropathy (such as a previous chronic kidney injury) when determining an appropriate time interval between the interventions. Therefore, there are currently many open questions regarding the optimal timing for these two procedures, especially in the light of conflicting results in several small studies.

**Figure 2:**
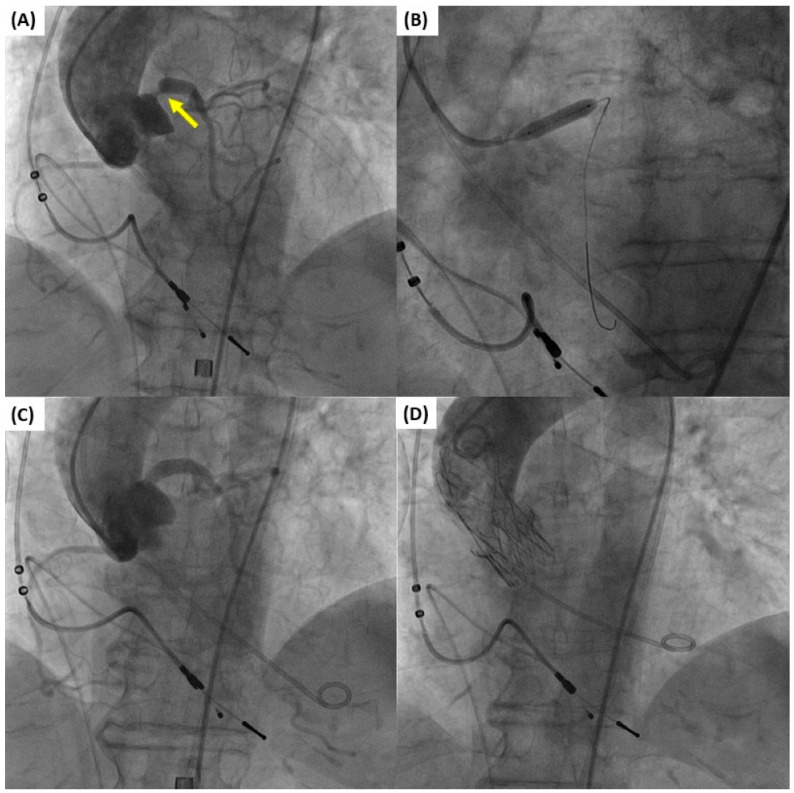
Simultaneous percutaneous coronary intervention (PCI) of the left main and transcatheter aortic valve implantation (TAVI) in an 80-year-old female patient with severe aortic stenosis and obstructive left main coronary artery disease (arrow). (**A**) Baseline aortogram showing aortic valve stenosis and critical stenosis of the left main coronary artery necessitating revascularization. (**B**) Stent implantation at the left main ostium. (**C**) Aortogram after PCI. (**D**) Aortogram after Accurate Neo2 (Boston Scientific, Natick, MA, USA) transcatheter aortic valve implantation.

A number of studies have supported the safety and feasibility of TAVI with concurrent PCI (Figure 2). Wenaweser et al. demonstrated that in a specific group of patients, revascularization by PCI can be safely performed alongside TAVI, either as a staged or simultaneous procedure [44]. Similarly, Conradi et al. presented their findings on the feasibility and safety of staged or single-stage TAVI and PCI in this high-risk patient population [45]. In addition, Pasic et al. demonstrated that the combined elective PCI and TAVI procedure using a single-stage technique is both possible and secure [46]. Conversely, Singh et al. demonstrated that patients who received PCI concurrently with TAVI experienced elevated rates of death while still in the hospital [47]. Similar findings were reported by Griese et. al, who found that performing contemporaneous PCI with TAVI in specific elderly individuals resulted in higher rates of early and late mortality [48]. While this approach has the benefit of avoiding multiple procedures and potentially lowering the risks associated with obtaining vascular access at different times, it does result in a higher volume of contrast media being used during the TAVI procedure. This could potentially raise the risk of contrast-induced nephropathy, especially in patients with complex CAD.

When considering the pathophysiological mechanisms that support staged PCI after TAVI, it is crucial to address AS as a priority. By correcting AS early on, we can reduce the excessive pressure on the LV, enhance microvascular circulation, and prevent temporary hemodynamic complications that could lead to renal or cerebral ischemia during PCI [49]. It is worth noting that the impact of the timing of PCI on clinical results may be influenced by several factors, such as the choice of THV, selection of vascular access route, and the dose of contrast medium used. These factors interact in a complicated manner with the therapeutic implications of PCI. However, it remains uncertain if there is a relationship between the timing of PCI and the level of revascularization [22,50]. Regarding the optimal timing, a dedicated analysis of the previously mentioned REVASC-TAVI registry provides some real-world evidence on this subject [22]. Among the available options for revascularization, the approaches of performing PCI before TAVI or simultaneously with TAVI were the most commonly used with a prevalence of 90.2%. However, even though the three groups (PCI before TAVI; PCI after TAVI; and PCI concomitant with TAVI) had similar levels of complexity and extents of CAD, a strategy of performing PCI after TAVI in a staged manner resulted in the most favorable clinical outcomes during the hospital stay and over a 24-month follow-up period, while performing PCI concomitant with TAVI, despite potential logistical advantages, appeared to have negative effects. Aside from the findings from the REVASC-TAVI registry, data regarding PCI following TAVI are limited. Van Rosendael et al. evaluated the clinical outcomes of patients who underwent PCI within one month versus more than one month before TAVI [51]. The study found no significant differences in total mortality between the two groups after a median follow-up of 24 months. Nevertheless, a notable rise in minor vascular and bleeding adverse events occurred in the group who underwent PCI within one month prior to TAVI. More evidence is required to elucidate which of the two strategies for patients with severe AS and associated CAD is better, PCI before TAVI versus PCI after TAVI. More evidence on this subject is expected to be available with the completion of the TAVI PCI trial (ClinicalTrials.gov ID NCT04310046), a randomized controlled trial providing a direct comparison of these two strategies [25,51].

**Table 2:** Summary of completed and ongoing trials comparing different treatment strategies for coronary artery disease revascularization in patients with severe aortic stenosis undergoing transcatheter aortic valve implantation.

First Author	Year	N of Patients	Study Groups	Study Type	Main Outcomes
Allali [20]	2016	17	PCI after TAVI	Retrospective	PCI after the implantation of an SEV CoreValve is usually feasible and safe but coronary access in an emergency setting can be challenging.
Conradi [45]	2011	28	PCI before or during TAVI	Clinical trial	Staged or single-stage TAVI + PCI proved feasible and safe in the high-risk population studied.
Faroux [52]	2020	1197	PCI before TAVI	Retrospective	Patients undergoing PCI pre-TAVI frequently exhibited complex coronary lesions and multivessel disease. PCI was most often efficient, and TLF and TVF rates at 2-year follow-up were low, also among patients with high-risk coronary features. However, overall MACCE occurred in about 1/3 of patients, with incomplete revascularization dictating an increased risk.
Kaihara [53]	2021	186	CAD involving the LM or proximal LAD lesion + PCI before TAVI vs. CAD not involving the LM or LAD proximal lesion + PCI before TAVI vs. CAD + TAVI	Retrospective	CAD with an LM or LAD proximal lesion are strong independent predictors of mid-term MACCEs and all-cause mortality in individuals with severe AS treated with TAVI. PCI before TAVI did not affect the outcome.
Kneizeh [54]	2022	21	PCI before or during TAVI	Retrospective	Temporary hemodynamic support with the Impella device during staged strategy with high-risk protected PCI appears to be safe and technically feasible in patients with severe AS pre-TAVI.
Kodra [55]	2023	276	PCI before or during TAVI	Retrospective	Complex and high-risk PCI can be safely executed in patients with complex CAD and severe AS.
Kumar [56]	2021	380	PCI before, during, or after TAVI	Prospective	Among patients who underwent concomitant PCI and TAVI, history of CABG, higher BMI, and statin pharmacotherapy had lower AE rates, while those discharged on warfarin had higher AE rates. AE rates were similar irrespective of timing of PCI.
Ochiai [57]	2020	258	PCI before, during, or after TAVI	Retrospective	There were no significant differences in terms of mid-term outcomes among pre-TAVI, concomitant, and post-TAVI PCI arms when the timing of PCI was carefully determined by heart team.
Pasic [46]	2012	46	PCI during TAVI	Prospective	The single-stage strategy with concomitant elective PCI and TAVI is feasible and safe. It has become the primary strategy choice for high-risk individuals with severe AS and CAD.
Santana [58]	2017	123	PCI before TAVI	Retrospective	In a select category of patients with concomitant CAD and AS, a combined strategy of PCI followed by minimally invasive AVR can be safely performed with very good short- and mid-term results.
Tran [59]	2022	5843	PCI with concomitant TAVI vs. PCI in the same hospitalization with TAVI vs. PCI in a subsequent hospitalization after TAVI	Retrospective	Concomitant PCI + TAVI was related with similar rates of in-hospital mortality but lower rates of AKI and lower resource utilization. While evaluating patient-specific factors, concomitant PCI + TAVI might be appropriate in selected individuals.
van Rosendael [51]	2015	96	PCI within 30 days before TAVI vs. PCI ≥30 days before TAVI	Retrospective	Shortly (<30 days) or remote (≥30 days) staged PCI pre-TAVI resulted in equivalent outcomes with the exception of minor vascular injury and minor bleeding events, which more frequently occurred in individuals treated with shortly staged PCI.
Wenaweser [44]	2011	256	CAD with PCI before TAVI vs. CAD with PCI with concomitant TAVI vs. TAVI without CAD	Prospective—Bern TAVI registry	CAD is recurrent among individuals with severe AS undergoing TAVI. Among carefully chosen patients, PCI can be safely performed besides TAVI, either as a staged or a concomitant intervention.
Beohar [60]	2022	18	Orbital atherectomy PCI before TAVI	Retrospective	Individuals with heavily calcified coronary lesions treated with orbital atherectomy pre-TAVI had low rates of MACE at 30 days and 12 months. The outcomes illustrate the feasibility and safety of orbital atherectomy for the management of complex calcific coronary lesions pre-TAVI.
Sabbah [61]	2023	455	Conservative management of CAD before TAVI vs. FFR-guided PCI before TAVI	NOTION-3 trial	Among individuals with CAD who were undergoing TAVI, PCI was associated with a lower risk of a composite of death from any cause, MI, or urgent revascularization at a median follow-up of 24 months than conservative treatment.
Stähli [15]	2024	986	Angiography-guided PCI after (within 1–45 days) TAVI vs. angiography-guided PCI before (within 1–45 days) TAVI	TAVI PCI Trial	Not completed yet.
Ribichini [16]	2024	320	Angiography-guided vs. physiology-guided PCI before, during, or after TAVI	FAITAVI trial	Not completed yet.

N of patients: number of patients, CAD: coronary artery disease, SS: SYNTAX score, TAVI: transcatheter aortic valve implantation, AE: adverse event, MI: myocardial infarction, SEV: self-expanding valve, PCI: percutaneous coronary intervention, AKI: acute kidney injury, AS: aortic stenosis, LM: left main, LAD: left anterior descending artery, SAVR: surgical aortic valve replacement, CABG: coronary artery bypass grafting, MACCE: major adverse cardiac and cerebrovascular events, MACE: major adverse cardiac events, FFR: fractional flow reserve, BMI: body mass index, TLF: target lesion failure, TVF: target vessel failure, AVR: aortic valve replacement, CAng: coronary angiography.

**Table 3:** Summary of completed studies comparing different strategies for coronary artery disease revascularization versus conservative treatment in patients with severe aortic stenosis undergoing transcatheter aortic valve implantation.

First Author	Year	N of Patients	Study Groups	Study Type	Main Outcomes
Abramowitz [62]	2014	249	PCI before TAVI vs. no-PCI	Retrospective	Performing PCI pre-TAVI in high-risk elderly individuals with significant CAD and severe AS is feasible and safe. It showed on escalation of the peri-procedural risk of AE or the all-cause mortality.
Rheude [50]	2023	1603	PCI before, concomitantly or after TAVI	REVASC-TAVI registry	In individuals with severe AS and stable CAD undergoing TAVI, performance of PCI post-TAVI seems to be connected with improved 24 months clinical results collated with other revascularization timing approaches.
Barbanti [63]	2017	136	PCI during TAVI vs. no-PCI	Prospective	In patients undergoing TAVI, screening of CAD with invasive CAng and ad-hoc PCI during TAVI is feasible and was not connected with increased peri-procedural risks. PCI followed by TAVI in the same session had similar results than TAVI with no-PCI.
Karaduman [64]	2021	127	PCI before, during, or after TAVI vs. no-PCI	Retrospective	Peri-procedural and long-term safety results and mortality rates are not significantly divergent between revascularized and non-revascularized individuals, and nor staged nor simultaneous PCI have AE in patients undergoing TAVI.
Valvo [65]	2023	302	PCI during TAVI vs. no-PCI	Retrospective	In individuals with severe AS + CAD, concomitant TAVI + PCI was as safe and effective as TAVI alone up to 36 months follow-up.
Patterson [39]	2021	235	PCI before TAVI vs. no-PCI	ACTIVATION Randomized Clinical Trial	Observed rates of death and rehospitalization at 12 months were similar between PCI and no-PCI pre-TAVI; nevertheless, the non-inferiority margin was not met, and PCI occurred in a higher percentage of bleeding.
Chakravarty [21]	2016	256	LM-PCI during TAVI vs. no-PCI	TAVR-LM registry	TAVI + LM-PCI is safe and technically feasible, with short- and intermediate-term clinical results analogous with those in individuals undergoing TAVI alone. These results suggest that TAVI + LM-PCI is a reasonable strategy for those who are at high risk from a surgical procedure.
Ghrair [66]	2020	1704	PCI during TAVI vs. no-PCI	Retrospective	In comparison to isolated TAVI, combined TAVI + PCI was related with a higher percentage of in-hospital morbidity and mortality.
Griese [48]	2014	411	PCI before or during TAVI vs. no-PCI	Prospective	Concomitant PCI is, when based on heart team decision, related with increased early and late mortality in selected elderly individuals undergoing TAVI.
Huczek [67]	2018	896	PCI before TAVI vs. no-PCI	Retrospective	Obstructive CAD at baseline assessment for TAVI has independent negative impact on short-term prognosis. Nevertheless, neither baseline nor residual SS values have prognostic ability in patients undergoing TAVI. PCI pre-TAVI seems to enhance survival to an equivalent extent as those without obstructive CAD at baseline.
Penkalla [68]	2015	593	CAD with PCI during TAVI vs. CAD without PCI during TAVI vs. TAVI without CAD	Prospective	Single-stage combined management of severe AS and highly relevant coronary lesions is a safe and feasible intervention. Early survival and survival up to 36 months are analogous to that observed in individuals presenting without CAD who received TAVI only. PCI effectively reduces the complexity of coronary lesions and even though more contrast agent is applied during the combined strategy, the rate of AKI was not elevated.
Benseba [69]	2023	1023	Angiography-guided PCI vs. angiography-guided no-PCI vs. FFR-guided PCI vs. FFR-guided no-PCI before TAVI	Retrospective	Intra-coronary adenosine is safe and well-received. There was no significant advantage to an FFR-guided approach related to an angiography-guided strategy regarding MACCEs. Even though clinically engrossing, abstaining from the procedural risks of PCI by postponing the procedure in functionally insignificant lesions failed to show a statistically significant benefit.
Shah [70]	2023	759	CAD with PCI before TAVI vs. CAD without PCI before TAVI vs. TAVI without CAD	Retrospective—VA CART	Among veterans undergoing TAVI, the presence of obstructive CAD is associated with higher mortality though coronary intervention before TAVI is not associated with improved results.
Tarantini [71]	2020	1936	Coronary access with or without PCI after TAVI	Retrospective—SOURCE 3 European Registry	Coronary access was needed at 36 months follow-up post-TAVI with a BEV in 3.5% of patients and was advantageous in all cases. The clinical success of PCI was 97.9%.
Zivelonghi [72]	2017	287	Optimal medical therapy vs. PCI angiography-guided before TAVI vs. PCI physiology-guided before TAVI	Prospective	The feasibility and safety of a physiology-guided revascularization approach was exhibited.

N of patients: number of patients, CAD: coronary artery disease, SS: SYNTAX score, TAVI: transcatheter aortic valve implantation, BEV: balloon-expandable valve, PCI: percutaneous coronary intervention, AKI: acute kidney injury, AS: aortic stenosis, LM: left main, LAD: left anterior descending artery, SAVR: surgical aortic valve replacement, CABG: coronary artery bypass grafting, MACCE: major adverse cardiac and cerebrovascular events, MACE: major adverse cardiac events, FFR: fractional flow reserve, BMI: body mass index, TLF: target lesion failure, TVF: target vessel failure, AVR: aortic valve replacement, CAng: coronary angiography.

## 6. Coronary Events After TAVI: Epidemiology and Pathophysiology

AS and CAD have overlapping risk factors, and several trials indicate a similar underlying physiological mechanism for both conditions [73]. Therefore, and considering the co-existence of the two conditions stated above, it is not surprising that coronary angiography and PCI may be needed in several patients after TAVI (Figure 1). The incidence of PCI after TAVI has been assessed by several observational studies, with an incidence of less than 3% in most cases. Weferling et al. conducted a study that revealed that 2.5–3.5% of patients who underwent TAVI also underwent coronary angiography following the operation, and PCI was performed in 27–55% of these cases [37]. In a study conducted by Nai Fovino et al. involving 912 individuals with a mean follow-up period of 2.1 years, the reported occurrence rate of coronary angiography was 5.3% [74]. The indication for angiography was ACS in 35% of instances, with PCI eventually performed in 26 individuals (54%), and an eventual incidence of PCI following TAVI of 2.8%. Younger age, prior PCI, and/or prior CABG were identified as factors that independently predicted the need for coronary angiography [21,52]. The SOURCE 3 Registry, which included 1936 patients treated with a SAPIEN 3 BEV, found that 3.5% of patients underwent coronary angiography during a 36-month follow-up. The main reasons for undergoing angiography were stable angina in 36.8% of cases, NSTEMI in 26.5% of cases, and STEMI in 11.8% of cases [75]. Among these patients, 69% underwent PCI, and the overall incidence of PCI was 2.4%. Similarly, Tarantini and colleagues observed a 2.4% PCI incidence after 3 years of follow-up [71].

Insufficient evidence exists about the frequency of coronary events following TAVI. Vilalta et al. observed an occurrence of acute coronary syndrome (ACS) in 10% of a group of 779 individuals who underwent TAVI [76]. This incidence was determined after a median follow-up period of about 2 years post-TAVI. The ACS occurrences were primarily composed of NSTEMI type 2 (36%), followed by unstable angina (35%), NSTEMI type 1 (28%), and STEMI (1%). Ultimately, a mere 39% of the patients received PCI. It is important to mention that the prognosis for ACS occurring after TAVI was unfavorable, with an all-cause mortality rate of 37% after an average follow-up period of 21 months following the occurrence of ACS. The majority of coronary events that occur following TAVI are most likely caused by an atherothrombotic mechanism. This can be attributed to the advancement of CAD or the failure of a PCI that was performed prior to the TAVI procedure. In addition, certain authors have proposed alternative mechanisms, including compromised blood flow dynamics and reduced blood supply to the coronary arteries due to the TAVI bioprosthesis [3,73,77,78]. Another possibility is the occurrence of a coronary embolism resulting from subclinical leaflet thrombosis in bioprosthetic aortic valves [79]. Late valve migration, which initially partially covers but does not completely block a coronary ostium, could also be a contributing factor. Lastly, a hypersensitivity reaction to metal anions present in the device (known as Kounis syndrome) is another potential mechanism that has been suggested [80]. Additional research is required to more accurately classify the specific kind and frequency of coronary events after TAVI, as well as to further examine the variables that contribute to the occurrence and unfavorable outcomes of patients who experience these events [81,82].

Furthermore, recent studies have highlighted the potential benefits of sodium–glucose cotransporter-2 (SGLT-2) inhibitors in patients with severe AS undergoing TAVI. A multicenter observational study by Paolisso et al. focused on diabetic patients with left ventricular ejection fraction (LVEF) < 50% and extra-valvular cardiac damage [83]. The study concluded that, over a 24-month follow-up, SGLT-2 inhibitor use was associated with a significant reduction in major adverse cardiovascular events (MACE), all-cause mortality, and heart failure hospitalizations. Specifically, these medications emerged as independent predictors of reduced MACE (HR: 0.45; 95% [CI] 0.17–0.75; *p*: 0.007), all-cause death (HR: 0.51; 95% CI 0.25–0.98; *p*: 0.042), and heart failure hospitalization (HR: 0.40; 95% CI 0.27–0.62; *p*: 0.004). These findings suggest that SGLT-2 inhibitors may offer cardioprotective effects in this high-risk population, potentially reducing coronary events post-TAVI. However, further large-scale RCTs are warranted to confirm these benefits and establish definitive clinical guidelines.

## 7. Procedural Considerations: Coronary Obstruction Risk and Management

The main procedural issues are mostly related to coronary obstruction [54,84]. This is considered one of the most concerning consequences of TAVI and is associated with a significant risk of death. Thankfully, the risk within procedures has been diminishing because of the advancements in pre-procedural imaging, patient selection, competence, and the availability of new-generation THVs. Occlusion can arise from several patho-mechanisms:

- Native leaflet obstruction;
- Sinus confiscation;
- Obstruction of the ostium by a mass within the leaflets;
- THV skirt or commissural impediment;
- Deformation and narrowing of a pre-existing ostial stent.

Both patient anatomy and THV variables contribute to the predisposition for coronary occlusion. Unfavorable anatomies refer to specific characteristics such as lengthy leaflets that are taller than the coronary ostial height, bulky leaflet calcification, coronary ostia that are positioned low, a deficient sinus of Valsalva, and a low sinotubular junction height. These factors are often assessed during pre-procedural CT imaging to identify individuals who are at risk [21,85]. The parameters that contribute to LV function include the height of the skirt and commissure as well as the height and type of the valve. Procedural considerations encompass the depth at which the valve is deployed and the subsequent inflation of the valve. Valve-in-valve operations have demonstrated an increased incidence of coronary blockage, most likely due to the displacement of the THV leaflets. Thorough study before a procedure can decrease the chances of obstruction by carefully selecting patients, complementing the shape of the aortic root to a suitable THV, and organizing the procedure to improve the visualization of the coronary arteries and the depth of the THV. For those who have a high risk of coronary blockage despite careful selection and planning, there are supplementary methods available to reduce this risk.

The choice of vascular access in patients undergoing PCI prior to TAVI holds significant implications, particularly in elderly populations with fragile vasculature. Traditionally, femoral access has been one possible route for PCI, especially when radial arteries are not suitable; however, this approach can complicate subsequent TAVI due to risks of vascular injury and access site limitations, especially in patients with severe peripheral arterial disease or calcification [30,86]. Recent evidence underscores the advantages of radial access, including distal radial access, which preserves the proximal radial artery for future interventions, reduces vascular complications, and improves patient comfort and recovery time [87,88].

The feasibility and safety of advanced procedures, such as distal radial balloon aortic valvuloplasty (DR-BAV), have further validated the utility of radial access [87,88]. This highlights the capability of the radial artery to accommodate complex procedures, even with larger sheath sizes. Moreover, recent studies advocate for radial-first strategies in PCI before TAVI, noting potential benefits in reducing procedural risks while maintaining vascular integrity for the subsequent valve intervention. Given the growing preference for minimally invasive approaches in elderly patients, radial access is increasingly recognized as the optimal strategy for PCI in TAVI candidates [87,88]. This approach not only preserves vascular access for TAVI but also facilitates innovations like radial-based valvuloplasty.

Coronary protection, which involves using a catheter and guidewire with or without a coronary balloon and stent, has been often used as a preventive measure for abrupt occlusion in high-risk vessels. It is recommended to perform this maneuver prior to the deployment of the THV because attempting to insert a catheter into the obstructed arteries after obstruction has a low likelihood of success. In addition, individuals experiencing these events frequently exhibit significant hemodynamic instability, making the implementation of a rescue technique rather difficult [21,40,89,90].

Chimney stenting is an intervention in which a coronary stent is initially positioned in a coronary artery and then quickly inserted and expanded if there is an obstruction [91]. The proximal stent edge is located inside the aorta, whereas the distal stent is positioned within the artery. A recent registry documented 60 cases, which accounted for 0.5% of the total 12,800 TAVI operations. The study monitored the results of these patients for a median duration of 612 days. Three patients passed away while hospitalized, and two instances of stent failure were documented after extended post-procedural monitoring [20,92,93]. The long-term consequences of this approach are still unclear.

Another alternative is the use of the BASILICA procedure, which involves intentionally creating a laceration in the THV or native aortic scallop to reduce the occurrence of iatrogenic coronary artery occlusion during TAVI [85,94,95]. This procedure is the deliberate use of an electrically charged coronary wire to cut and tear the valve leaflets. It is most effective for treating individuals who are at risk of coronary blockage, either due to a leaflet directly blocking the artery or indirectly due to the aortic sinus being blocked. This procedure may be performed on both natural aortic valves and THVs. The procedure essentially consists of cutting the leaflet responsible for the event from the bottom to the top, causing the split leaflet to be positioned on either side of the coronary ostium. The BASILICA study in 2019, with a cohort of 30 participants, demonstrated a 95% success rate in traversing and lacerating, with no instances of coronary obstruction. Furthermore, a recent multicenter registry, with 214 patients, demonstrated a high success rate of around 94% in terms of leaflet traversal and laceration. Procedural success was defined as the successful completion of the procedure without death, occlusion of the coronary arteries, or the need for emergency operation. This was achieved in 86.9% of the patients, and out of them, 4.7% experienced partial or total occlusion of the coronary artery despite undergoing this procedure. Out of the total of ten patients who were impacted, six experienced partial occlusion that was successfully alleviated with the use of stenting. Among the four cases with total occlusion, a single patient had relief of the right coronary artery occlusion with balloon angioplasty. Out of the three remaining cases with total obstruction of the LM coronary artery, one was treated by chimney stenting, while the other two required THV snaring and the placement of a fresh THV [27,60,92].

A dedicated leaflet modification device, the ShortCut, was recently introduced for use prior to TAVI in patients at risk of coronary artery obstruction [96]. Its safety and efficacy were tested in 60 patients, with 100% procedural success [100%; 95% (CI): 94–100.0%, *p* < 0.001]. Additionally, there were no deaths, and only one (1.7%) stroke, suggesting that this device was safe and may yield favorable clinical results in individuals at risk of coronary obstruction undergoing TAVI [96].

## 8. Coronary Access After TAVI

Life expectancy following the TAVI procedure has increased, as it is increasingly being performed on younger individuals. Coronary re-access is becoming crucial in this patient population due to the potential occurrence of future acute or chronic coronary syndromes, with a high incidence and a high mortality rate, as discussed above. Therefore, in anticipation of the need for future invasive coronary angiography in a number of patients, strategies to facilitate future catheterization should be considered. When scheduling a TAVI procedure, it is crucial to take into account the patient’s long-term coronary journey. This is particularly true considering the probable occurrence of future TAVI-in-TAVI operations. Choosing the right first THV is crucial to prevent future complications. Prior research has examined the feasibility of performing coronary angiography after TAVI for both self-expanding and balloon-expandable THVs [10,97]. The success rates for most cases were above 90%, indicating a high level of effectiveness. Additionally, the success rates for PCI have also been shown to be high, as well. Due to anatomical factors, performing angiography on the right coronary artery may provide additional challenges compared to the left. However, the success rates remain high. Small trials investigating PCI of the LM coronary artery following TAVI have also demonstrated favorable success rates. Various guidelines exist to facilitate cannulation of the coronary ostia, taking into account the AoRo architecture and the THV frame. Cannulation can be achieved by either passing through the stent cells or by accessing the sinus from a position above the THV frame. The use of coronary wires, balloons, or guide extension catheters may be required [24,98,99,100,101]. 

In terms of selecting THVs, self-expanding valves have an inflow that firmly attaches the frame to the annulus, a concave section in the center, and a sizable outflow that sits in the ascending aorta. Self-expanding valves come in various designs, and those with bigger cell sizes provide better access to the coronary region. The concave section in the center decreases the likelihood of coronary obstruction by increasing the volume in the sinuses of Valsalva. The height of the skirt may impede the coronary ostia in a sudden manner and also inhibit future re-access. However, this can be reduced by managing the depth of implantation [97]. However, the presence of THV commissure posts might also hinder the ease of accessing the coronary arteries if they align with the coronary arteries’ ostia. Balloon-expandable THVs lack a central concavity, unlike self-expandable THVs, and possess a shorter stent frame. The use of a shorter stent frame enables easier access to the coronary sinus by allowing catheters to enter above the frame, rather than through the stent cells. CT-based investigations have indicated that balloon-expandable THV frames are rarely positioned above the coronary ostia. Among these individuals, there was no statistically significant effect observed on the incidence of early MI or on the occurrence of the need for future PCI [30]. This phenomenon has been hypothesized to be linked to the presence of sinuses with enough depth, which prevents the coronary ostia from being directly aligned with the THV. Post-procedural CT imaging can assist in determining the best approach for coronary access in stable individuals. However, its use may be restricted in patients with ACS. Imaging studies have demonstrated that in SAVR, bioprosthetic THV commissures are frequently aligned with the native commissures and the coronary ostia. However, in TAVI, the alignment of neo-commissures with coronary ostia is often unpredictable, leading to a significant concern regarding commissural post-procedural overlap [10].

Various methods have been developed to effectively align neo-commissures with the coronary ostia using commissural alignment techniques. One strategy includes manipulating the location of the delivery catheter prior to insertion via the femoral artery, and then making precise adjustments while the catheter is in the descending aorta or AoRo [102]. Although several methods have demonstrated efficacy in enhancing alignment, modern technologies nevertheless possess notable constraints. In the ALIGN-TAVR study, 24.3% of individuals using the Evolut THV and 12.5–14.3% of patients using the ACURATE neo THV still experienced neo-commissural overlap with one or both coronary arteries even after optimization. Efforts to compress the Sapien 3 THV at a consistent commissural orientation for improvement did not result in a change in the occurrence of the overlap [99]. A new study on CT-based optimization of coronary alignment indicates that aligning coronary arteries may have an advantage over aligning commissures.

## 9. Future Directions

There are currently several deficiencies in the data regarding the management of CAD in individuals undergoing TAVI. The efficacy of revascularization prior to TAVI will be more definitively determined in future randomized studies (Table 2). The quantification of ischemia may have a significant impact on determining the need for revascularization, similar to its significance in CAD in general. In terms of procedural management, THVs are continuously developing and enhancing their design. Enhanced understanding of the elements that impact coronary obstruction has led to significant advancements in newer-generation THVs, resulting in reduced procedural risks. It is probable that more advanced procedures will develop to enable a higher probability of success in accessing the coronary arteries. As TAVI advances, there is a growing recognition that patients are more likely to require TAVI-in-TAVI operations in the future. The use of valve-in-valve operations will lead to an increased risk of coronary occlusion and difficulties in accessing the affected area. Future research would ideally provide insight into an optimal approach for younger patients who will need to endure numerous operations [10].

## 10. Conclusions

CAD is frequently observed in individuals who undergo TAVI. The intricate interaction between CAD and TAVI encompasses the connections among the patient’s risk profile, the structure of the aortic root, the design and placement of the THV, and the methods employed to prevent coronary problems. An extensive and sophisticated comprehension of CAD management in TAVI patients is crucial for maximizing patient outcomes, not only in the short term but also over their anticipated lifespan. While studies have generally not demonstrated a positive therapeutic effect of regularly undergoing coronary revascularization in patients receiving TAVI with concurrent CAD, current guidelines nevertheless advise PCI for TAVI candidates who have substantial CAD. Although the current ACC/AHA guidelines advocate for performing PCI prior to TAVI, the European guidelines do not provide a precise recommendation about the proper timing. The optimal timing for performing PCI in candidates for TAVI is now a subject of continuing discussion and requires further research.

The ongoing TAVI PCI study is necessary to provide clarity on the most suitable approach and timing. Theoretically, the unobstructed access to the coronary arteries, the decrease in the amount of heart muscle damage before TAVI, and the lower quantity of contrast used when performed in two separate sessions (staged operations) suggest a potential advantage of the approach to perform revascularization before TAVI. Currently, the care of CAD in patients who are candidates for TAVI should be tailored to each person by the heart team, considering factors such as the patient’s age, comorbidities, coronary architecture, and the severity of each lesion.

## Figures and Tables

**Table 1:** Summary of completed trials comparing different treatment options for coronary artery disease revascularization, including surgical and percutaneous treatment options, in patients with severe aortic stenosis undergoing transcatheter aortic valve implantation.

First Author	Year	N of Patients	Study Groups	Study Type	Main Outcomes
Baumbach [41]	2019	626	SAVR + CABG vs. TAVI + (off-pump/minimally invasive coronary artery bypass—OP/MIDCAB) vs. TAVI + PCI	Prospective	TAVI + OP/MIDCAB patients share many characteristics with TAVI + PCI patients, with only slightly poorer long-term outcomes. In patients ineligible for SAVR + CABG and TAVI + PCI, hybrid interventions are reasonable second-line options.
Alperi [40]	2021	312	TAVR + PCI vs. SAVR + CABG	Prospective	TAVI + PCI and SAVR + CABG were associated with similar rates of MACCE after a median follow-up period of 3 years but TAVI + PCI recipients exhibited a higher risk of repeat coronary revascularization.
Søndergaard [42]	2019	1660	TAVR + PCI vs. SAVR + CABG vs. TAVI without CAD vs. SAVR without CAD	Prospective—SURTAVI trial	For patients at intermediate surgical risk with severe AS and non-complex CAD (SS ≤ 22), a complete percutaneous approach of TAVI and PCI is a reasonable alternative to SAVR + CABG.

N of patients: number of patients, CAD: coronary artery disease, SS: SYNTAX score, TAVI: transcatheter aortic valve implantation, PCI: percutaneous coronary intervention, AKI: acute kidney injury, AS: aortic stenosis, LM: left main, LAD: left anterior descending artery, SAVR: surgical aortic valve replacement, CABG: coronary artery bypass grafting, MACCE: major adverse cardiac and cerebrovascular events, MACE: major adverse cardiac events, FFR: fractional flow reserve, BMI: body mass index, TLF: target lesion failure, TVF: target vessel failure, AVR: aortic valve replacement, CAng: coronary angiography.
